# Can a physiotherapy student assume the role of an advanced practice physiotherapist in Orthopaedic surgery triage? A prospective observational study

**DOI:** 10.1186/s12891-019-2864-x

**Published:** 2019-10-29

**Authors:** David Yin, François Cabana, Yannick Tousignant-Laflamme, Sonia Bédard, Michel Tousignant

**Affiliations:** 1 Université de Sherbrooke, 3001, 12e Avenue Nord, Sherbrooke, Québec Canada; 2 CIUSSS de l’Estrie-CHUS, 3001, 12e Avenue Nord, Sherbrooke, Québec Canada; 3 Centre de recherche du CHUS, 3001, 12e Avenue Nord, Sherbrooke, Québec Canada; 4 Centre de recherche sur le vieillissement, 1036 Rue Belvédère Sud, Sherbrooke, Québec Canada

**Keywords:** Accessibility, Inter-rater agreement, Diagnosis, Management, Satisfaction, Physiotherapy, Orthopaedics

## Abstract

**Background:**

Advanced practice physiotherapists (APP) have helped improve accessibility to orthopaedic outpatient care. Several studies have validated the APP practice model in orthopaedic care, demonstrating high agreement between APPs and orthopaedic surgeons (OS) regarding diagnosis and management. However, as APPs tend to be experienced senior physiotherapists, such a study involving physiotherapy students (PS) has not yet been explored. The objective of this study was to evaluate the agreement for orthopaedic diagnoses and surgical triage between a PS and OSs.

**Methods:**

A prospective study involving a final year PS and seven OSs was conducted in a university hospital, after the PS had undergone a three-week intensive training. Eighty-six adult patients referred to OSs for knee osteoarthritis, hip osteoarthritis or shoulder problem were independently evaluated by the PS, and then re-evaluated by an OS. The diagnoses and surgical triage recommendations of both clinicians were analyzed for agreement using raw percent agreement and Cohen’s kappa. Patient satisfaction with the outpatient clinic experience was noted using a modified version of the Visit-Specific Satisfaction Instrument.

**Results:**

Our sample consisted of 86 patients (mean age = 63.4 years). Reasons for consultation included shoulder problems (36%), knee osteoarthritis (52%) and hip osteoarthritis (12%). The raw percent agreement for diagnosis was 95.3%. The agreement for surgical triage was high (κ = 0.86, 95% CI: 0.74–0.98) with a raw agreement of 94.2%. Patient satisfaction was high.

**Conclusions:**

The PS and OSs made similar diagnoses and triage recommendations suggesting that clinical experience alone is not a prerequisite for physiotherapists to help increase accessibility to orthopaedic care.

## Key points

- The physiotherapy student was capable of making similar diagnoses and surgical triage recommendations as orthopaedic surgeons.
- Patients were highly satisfied with the outpatient clinic practice model involving a physiotherapy student collaborating with orthopaedic surgeons.
- The role of advanced practice physiotherapist in orthopaedic surgery should not be limited to experienced senior physiotherapists, which could possibly lead to improved accessibility to orthopaedic care.
- However, the study is limited by the evaluation of a single physiotherapy student in a single clinical setting.

## Background

Access to orthopaedic care can be challenging for Canadians [1–3], as wait times for orthopaedic clinic referrals can sometimes exceed 2 years [2]. The shortage of orthopaedic surgeons (OS) means that the current landscape of orthopaedic medicine in Canada is plagued by work overload [3, 4]. Moreover, inadequate management and referrals by general practitioners lead to unnecessary consultations by OSs [5], which enhances the problem. Also responsible for the limited access to care is the current practice model in orthopaedic medicine which is centered on the physician.

Providing assistance to OSs would help improve accessibility while reducing workload for these surgeons. In some countries, a new model of practice involving physiotherapists in orthopaedic outpatient clinics has proved to be effective [6, 7]. Physiotherapists working in these new practice models are often referred to as advanced practice physiotherapists (APP). Their primary role is to triage for surgical conditions. Tasks frequently performed by orthopaedic APPs include evaluating initial consultations, making diagnoses, ordering laboratory tests and imaging and ensuring follow-up of non-surgical conditions [2, 7]. APPs, though scarce, have been successfully implemented in numerous countries [7–13]. Several studies examining agreement of clinical diagnosis and surgical triage between APPs and OSs show that APPs can establish similar diagnoses as OSs for a variety of musculoskeletal problems, with raw percent agreement ranging from 75 to 92% [10, 11, 13, 14]. Strong agreement (86–92%; κ = 0.69–0.80) has also been shown for triaging surgical patients [10–14]. Furthermore, orthopaedic clinics involving APPs have generated high satisfaction [8–11, 13, 15]. Thus, APPs are well suited for seeing new orthopaedic consultations in an outpatient setting.

All current studies evaluating APPs in orthopaedic outpatient clinics have involved senior physiotherapists with many years of experience [16]. No study has been conducted on a collaborative practice model involving junior physiotherapists. Therefore, the primary objective of this study was to investigate the level of agreement on surgical triage between a physiotherapy student (PS) and OSs working in a collaborative outpatient clinic. As secondary objectives, the level of agreement between these clinicians on clinical diagnoses and patient management was assessed as well as patient satisfaction with the collaborative practice model.

## Methods

### Design

A prospective inter-rater reliability study of consecutive cases was conducted during a 4 week period (September 2017 – October 2017) in the orthopaedic outpatient hospital clinic of the Centre Hospitalier Universitaire de Sherbrooke (CIUSSS de l’Estrie-CHUS), Quebec, Canada, a tertiary university hospital.

### Participants

New patients above 18 years of age referred to our orthopaedic outpatient clinic for issues related to knee osteoarthritis, hip osteoarthritis or shoulder problems were eligible. Patients were excluded if they did not comprehend French or English, or if they were unable to give an informed consent.

Consecutive admissible patients on the waiting list for an orthopaedic consultation were contacted and recruited for the study by a research assistant. Informed written consent was obtained the day of their appointment. The project was approved by the local Ethics Review Board. The rights of the participants were protected.

### Physiotherapy student

A 23 year old male PS in his last year of a master’s degree (520 h of clinical experience) was selected to participate and integrate into our orthopaedic outpatient clinic during a seven-week clinical rotation. Selection was not related to academic performance. Prior to the start of the study, the PS underwent a three-week intensive training with the orthopaedic team in order to become familiar with the APP role. The training included shadowing OSs, attending review sessions on high-yield topics, practicing clinical evaluations of patients and receiving constant feedback on performance from OSs and residents.

### Orthopaedic surgeons

Seven senior OSs from varying subspecialties participated in our study. All of the OSs have fellowship training and at least 10 years of experience. They work at teaching hospitals where many orthopaedic surgery residents and medical students receive training under their supervision.

### Collaborative model in the outpatient clinic

A collaborative model of APP-led orthopaedic outpatient clinic was implemented at our hospitals for the purpose of the study. The collaborative model involved a preliminary evaluation (patient history, physical examination and imaging interpretation) of new consultations by an APP and revision of each patient with an attending OS. The OS then personally assessed the same patient to determine the final clinical management to be prescribed during that same visit. The role of the APP was assumed by the PS in our study. All patients were recruited prior to their appointment and informed that they would be assessed by a PS before seeing their OS.

### Data collection and procedures

Socio-demographic characteristics of each participant as well as the referring family physician’s management of the participant’s condition prior to their consultation were recorded including previous medication, infiltrations, imaging and therapy. Based on this information, the OSs determined the appropriateness of the initial management received by each participant. An initial management was considered ideal when the referring physicians had performed all possible treatment modalities within their scope of practice.

As per the collaborative model, both the PS and OS evaluated each participant independently. A standardized data collection form was created to collect each clinician’s clinical decisions. The primary outcome was their surgical triage recommendation and was noted as either “conservative” or “surgical” treatment. The patients’ most likely primary clinical diagnosis was recorded.. Additional management suggestions were noted as well. These included further imaging and conservative treatment modalities. The clinicians also decided whether a follow-up with the patient’s family doctor or OS was most appropriate.

The PS completed the data collection form immediately after his evaluation, prior to reviewing with the OS. The OS was blinded to the recommended clinical decisions of the PS. The clinical decisions of the OSs were considered the gold standard to which those of the PS were compared to.

Finally, patients’ satisfaction with their outpatient clinic visit was assessed with a modified version of the Visit-Specific Satisfaction Instrument (VSQ-9) [15]. The first two items of the questionnaire (“Getting through to the office by phone” and “Length of waiting time at the office”) were not included in the analysis as they were not directly related to the collaborative outpatient clinic model. Participants answered the VSQ-9 immediately following their discharge and their responses kept confidential from their clinician.

### Analyses

The participants’ clinical characteristics and satisfaction score were analyzed using descriptive analysis. Raw percent agreement and Cohen’s kappa with 95% confidence interval (95% CI) were used to measure clinical decisions agreement between the PS and OS. The strength of agreement using kappa (κ) was interpreted as suggested by Landis et al.: 0.0–0.20 = slight agreement, 0.21–0.40 = fair, 0.41–0.60 = moderate, 0.61–0.80 = strong and 0.81–1.00 = almost perfect [17]. Kappa ≥0.41 was considered clinically significant. Three senior OSs reviewed the clinical diagnoses recorded by the PS and OSs to determine agreement. The OSs were blinded as to whom made the diagnosis. The initial inter-rater concordance between the three OS reviewers judging diagnostic agreement between the PS and OSs was good (κ = 0.65–0.88). Differences between the reviewers were resolved through consensus.

Sample size was calculated based on triage agreement according to the method proposed by Flack et al. [18] An alpha threshold of 5%, power of 80% and bilateral test were used in the calculation. In a study conducted by Desmeules et al [11], the proportion of patients deemed surgical was 30.8%. The expected kappa was chosen to be 0.70 according to what was found in the literature [11–13]. A theoretical kappa of 0.40 was chosen. Accordingly, a sample size of 75 patients was needed.

### Source of funding

A research grant for masters training was obtained from the Foundation for research and teaching in orthopaedic surgery of Sherbrooke. There were no other sources of funding.

## Results

**Fig. 1:**
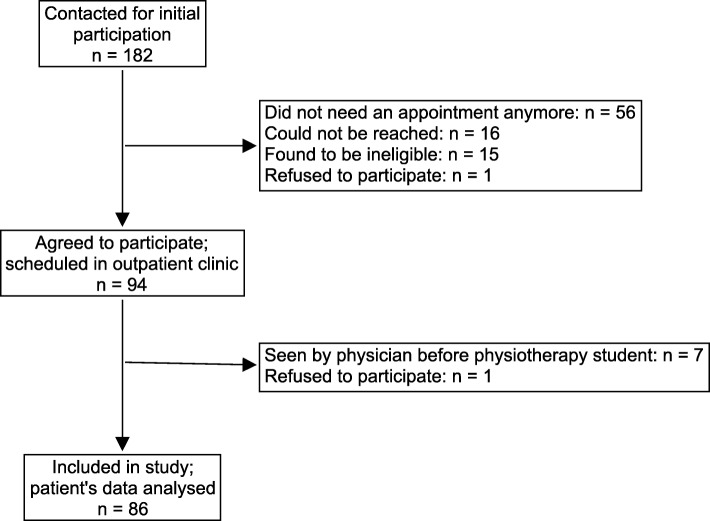
Flow chart of patient recruitment

Out of the 182 patients contacted for initial participation in the study, 86 were seen by the PS and included in the study. Complete flow chart of recruitment is detailed in Fig. 1.

### Clinical characteristics

Our sample was composed of 86 patients (60% male, 40% female), with an average age of 63.4 years (95% CI: 41.4–85.4). Reasons for consultations included shoulder problems (36%), knee osteoarthritis (52%) and hip osteoarthritis (12%). As for flow of referral, 43% of patients presented during the first half of the study and 57% during the last half.

The family physician management provided to the patients prior to consultation is presented in Table 1. Oral medication was prescribed to 88% of participants and a topical anti-inflammatory cream to 8%. An appropriate corticosteroid infiltration was performed in 63% of patients. Almost all participants (95%) had undergone some form of imaging while 45% had tried a form of non-pharmaceutical therapy (physiotherapy was the most observed at 37%). According to the OSs, only 58% of participants had received an ideal management of their orthopaedic problem before their consultation.

**Table 1:** Previous conservative management of participants

Management Modality	Frequency	Percent
Oral Medication	76	88%
Non-opioid analgesics	71	83%
Opioid analgesics	11	13%
NSAIDs	40	47%
Pregabalin	4	5%
Corticosteriods	2	2%
Muscle relaxant	2	2%
Amitriptyline	1	1%
Topical Medication		
Topical NSAIDs	7	8%
Appropriate corticosteroid infiltration	54	63%
Imaging	82	95%
X-ray	81	94%
CT-scan	1	1%
MRI	23	27%
Ultrasound	3	4%
Bone scintigraphy	1	1%
Non-Pharmacological Therapy	39	45%
Physiotherapy	32	37%
Other including CAM^a^	8	9%

*CAM* complementary and alternative medicine

^a^includes chiropractic, kinesiology, massotherapy, occupational therapy, orthotherapy, osteopath

### Clinical decisions agreement

#### I. Clinical diagnosis

The distribution of the clinical diagnoses encountered during the study is listed in Table 2. Raw percent agreement of clinical diagnosis made by the PS and OSs was 95.3%. Shoulder problems contributed to three of the PS’s misdiagnoses and knee problems for one.

**Table 2:** Primary clinical diagnoses of participants (after validation process by three OS)

Clinical Diagnosis	Frequency	Percent
Knee Osteoarthritis	34	39.5%
Rotator Cuff Tear	13	15.1%
Hip Osteoarthritis	9	10.5%
Glenohumeral Osteoarthritis	5	5.8%
Uni-compartmental Knee Osteoarthritis	5	5.8%
Shoulder Impingement	4	4.7%
Acromioclavicular Osteoarthritis	3	3.5%
Anterior Shoulder Instability	3	3.5%
Rotator Cuff Arthropathy	3	3.5%
Meniscal Tear	2	2.3%
Patellofemoral Osteoarthritis	2	2.3%
Baker’s Cyst	1	1.2%
Patellofemoral Pain Syndrome	1	1.2%
Trochanteric Bursitis	1	1.2%

#### II. Surgical triage

The PS and OSs agreed on surgical triage outcomes for 94.2% of cases (κ = 0.86; 95% CI: 0.74–0.98). The OSs considered 31.4% of participants required surgery (Table 3a). The five discordant cases were further analyzed (Table 4).

**Table 3:** Inter-examiner agreement for surgical triage for a) all patients, b) patients seen during first half of study and c) patients seen during second half of study

a)			
Surgical Triage		Orthopaedic Surgeons	
		Conservative	Surgical
Physiotherapy Student	Conservative	58	4
	Surgical	1	23
Raw percent agreement = 94.2%; κ = 0.86 (95% CI: 0.74–0.98)			
b)			
Surgical Triage Week 1 + 2		Orthopaedic Surgeons	
		Conservative	Surgical
Physiotherapy Student	Conservative	28	3
	Surgical	0	6
Raw percent agreement = 91.9%; κ = 0.75 (95% CI: 0.49–1.00)			
c)			
Surgical Triage Week 3 + 4		Orthopaedic Surgeons	
		Conservative	Surgical
Physiotherapy Student	Conservative	30	1
	Surgical	1	17
Raw percent agreement = 95.9%; κ = 0.91 (95% CI: 0.79–1.00)			

κ: Cohen’s kappa; 95% CI: 95% confidence interval

**Table 4:** Detailed descriptions of discordant cases for surgical triage

Case	Clinical diagnosis	Surgical triage	Reason for wrong surgical triage
1	Hip osteoarthritis	Surgical	First patient seen during study period; PS made same diagnosis as OS, however deemed condition to be of lesser severity when interpreting X-rays; PS suggested a conservative treatment.
2	Glenohumeral osteoarthritis	Surgical	Second patient seen during study period; PS made wrong diagnosis of rotator cuff tear due to misinterpretation of x-rays; PS suggested a conservative treatment.
3	Patellofemoral osteoarthritis	Surgical	PS made wrong diagnosis of patellofemoral pain syndrome due to inaccurate physical examination of patient; PS suggested a conservative treatment
4	Rotator cuff tear	Surgical	PS made same diagnosis as OS, however was unaware of existence of a rarely performed new surgical intervention (arthroscopic superior capsule reconstruction for irreparable rotator cuff tears); PS suggested a conservative treatment due to perceived inability to operate patient.
5	Knee Osteoarthritis	Conservative	PS made same diagnosis as OS; Patient had previously undergone a meniscectomy on same knee and developed knee osteoarthritis afterwards; PS deemed the condition to be of greater severity; PS suggested a surgical treatment.

*PS* physiotherapy student, *OS* orthopaedic surgeon

Comparison of surgical triage agreement from first half of the study period (Table 3b) to second half (Table 3c) showed improvement with time, where raw percent agreement increased from 91.9 to 95.9% while agreement improved from strong (κ = 0.75; 95% CI: 0.49–1.00) to almost perfect (κ = 0.91; 95% CI: 0.79–1.00).

#### III. Additional imaging and conservative treatment recommendations

Additional management recommendations (treatment or imaging) varied between the PS and OSs. Generally, the PS suggested less additional imaging tests compared to the OSs (PS: 15.1% vs. OS: 30.2%) and agreement for these was moderate (κ = 0.45). X-rays were the most frequently recommended imaging modality by both the PS and the OS. The majority of patients were deemed requiring some form of conservative treatment by both clinicians (PS: 76.7% vs. OS: 82.6%). Agreement between the PS and OSs for prescribing a conservative treatment modality was weak (κ = 0.39). These treatment modalities included advice and education, medication adjustments, local corticosteroid infiltration, orthotics, walking aid, exercises and outpatient physiotherapy referral. Detailed results are presented in Table 5.

**Table 5:** Additional imaging and conservative treatment recommendations made by the physiotherapy student and orthopaedic surgeons

Further Management	Physiotherapy Student		Orthopaedic Surgeons		Agreement (κ)
	Frequency	Percent	Frequency	Percent	
Imaging	13	15.1%	26	30.2%	0.45
X-Ray	9	10.5%	19	22.1%	0.50
CT-Scan	3	3.5%	5	5.8%	0.74
MRI	0	0.0%	1	1.2%	0.00
Other Imaging^a^	1	1.2%	1	1.2%	N/A
Conservative treatment modalities	66	76.7%	71	82.6%	0.39
Advice and Education	43	50.0%	43	50.0%	0.26
Adjustments to Medication	24	27.9%	20	23.3%	0.39
Local Corticosteroid infiltration	44	51.2%	40	46.5%	0.49
Orthosis or Walking Aid	5	5.8%	13	15.1%	0.39
Exercises	35	40.7%	24	27.9%	0.47
Outpatient Physiotherapy Referral	11	12.8%	13	15.1%	0.32

N/A: not applicable. ^a^ included quantitative computed tomography (physiotherapy student) and ultrasound (orthopaedic surgeon)

#### IV. Most appropriate physician to assume follow-up

Agreement on which physician was most appropriate to assume patient follow-up was almost perfect (κ = 0.81; 95% CI: 0.69–0.94) with a raw percent agreement of 90.7%. Follow-up with an OS was deemed necessary in 43.0% of patients.

### Patient satisfaction

Patient satisfaction with the outpatient clinic model was high with an average total score of 90.0% (95% CI: 67.5–100%). Mean time taken by the PS to perform his evaluation was 30.9 min. (95% CI: 18.5–43.3 min.).

## Discussion

Our study results show that a physiotherapy student (PS) in his final year of studies was capable of making similar diagnoses and surgical triage recommendations as an orthopaedic surgeon (OS) for cases of knee osteoarthritis, hip osteoarthritis or shoulder problems.

Strong agreement was observed for diagnoses made by the PS and OSs. The PS was capable of differentiating between more nuanced orthopaedic entities such as “knee osteoarthritis vs. uni-compartmental knee osteoarthritis” and “glenohumeral osteoarthritis vs. rotator cuff arthropathy”. The PS’s ability to correctly diagnose orthopaedic entities was similar to that of APPs in the current literature which reported diagnostic raw percent agreement varying between 69 and 98% [10, 11, 13, 14]. Furthermore, compared to similar studies, a greater variety of orthopaedic problems, spanning three joints, was seen by the PS.

Disagreements between the PS and OSs on clinical diagnosis were limited to a few cases. Most of the PS’s misdiagnoses came from patients presenting shoulder problems. Referrals for shoulder problems were generally more challenging as the PS was required to make larger differential diagnoses. The clinical diagnoses of the OSs were considered the gold standard. It is also possible that some of the OS’s diagnoses may have been incorrect, meaning discordant cases may not have resulted from incorrect diagnosis by the PS. Nevertheless, diagnostic agreement was very high.

The main role of APPs has involved determining surgical candidates from orthopaedic consultations. The PS has demonstrated exceptional proficiency in surgical triage. His performance was comparable to those of APPs who also reported strong agreement with OSs for surgical triage [10–14]. A learning curve was observed as surgical triage agreement improved from strong during the first 2 weeks to almost perfect over the last 2 weeks of the 4 week study period. However, it should be noted that there exists an overlap of 95% confidence intervals between those two periods. Disagreements in surgical triage may be attributed to the PS’s lack of experience. Half of these cases were encountered early during the study while the PS was still adapting to his role and the other half were more complex cases requiring advanced knowledge of orthopaedic medicine.

The PS and OSs disagreed more often when it came to recommending additional management modalities. Desmeules et al. also explored further imaging and management recommendations made by APPs. For imaging, they found highest agreement in computed tomography scan (CT-scan) prescription. This could be attributed to the existence of well-defined criteria for CT-scan use. The lowest agreement was in X-ray prescription and the APP prescribed X-rays less than the OSs [11], similar to what was obtained in our study. While imaging may be necessary for establishing diagnoses, it can also be used to plan surgeries. This differentiation was not specified in our study and therefore may have accounted for the under-prescription of X-rays by the PS.

Desmeules et al. also observed that their APP prescribed more conservative treatment modalities than OSs [11]. This was not the case with our PS. A possible cause for the discrepancies in management could result from the different preferences of the many OSs involved. Moreover, considering the short training period of the PS, he may not have had enough time to acquire adequate knowledge on medication use. Perhaps counting treatment modalities prescribed by each clinician is not an effective way to collect data for this purpose.

The PS and the OSs agreed strongly on the most appropriate physician to assure patient follow-up. The majority of patients (57.0%) did not require follow-up with an OS, close to the proportion of patients that did not obtain an ideal management of their problem prior to consultation (58.1%). It should be noted that 68.6% of patients in the study did not require a surgical intervention as is similarly reported in previous studies [2, 13]. OSs are physicians trained extensively in performing surgeries for musculoskeletal problems. The fact that most patients referred in orthopaedics are not surgical candidates and have not been properly managed by their family physicians may be the most concerning contributor to the lack of accessibility in this specialty. However, some family physicians might be less confident with musculoskeletal problems as they deal with more complex global medical management of their patients. Thus, the implementation of more orthopaedic clinics involving APPs seems to be a logical and viable solution.

Finally, patients were overall satisfied with the clinical practice model involving a PS assisting OSs. The VSQ-9 scores obtained are comparable to those from APP-led clinics which also reported excellent satisfaction scores [8–11, 13, 15]. It has been shown that patient satisfaction correlates with time spent with a clinician [19, 20], which could explain the high satisfaction with APP-led clinics. Furtthermore, high patient satisfaction correlates with improved health outcomes such as better treatment adherence [21]. Very few patients refused being seen by the PS, thus demonstrating the acceptability of such a practice by patients.

Currently, no formal training exists for physiotherapists who desire to become APPs [2]. In order for APPs to become more prevalent in orthopedic medicine and have positive impacts on accessibility to care, a standardized, recognized training program needs to be established. Our study supports starting such training during a physiotherapist’s education. More experience with PSs in the APP role would further strengthen this statement. Future research can explore the process of creating a formal APP training program for PSs.

### Strengths and limitations

One of the strengths of the current study is that it is the first of its kind to evaluate a PS in the role of an APP in an orthopaedic outpatient setting. Unlike other studies conducted on APPs [10–14], the PS was not limited to a single type of orthopaedic subspecialty, but saw a wider variety of conditions spanning three major joints. Another strength of the study was that the PS worked with seven different OSs, more than any other study on APPs currently found in the literature. Comparing the PS to multiple OSs allowed for more generalizable results. Chances are that the different OSs had varying preferences for managing their patients, and due to this, agreement between the PS and OSs seems unlikely to have been over-estimated.

Our study was limited by having only one PS in one clinical setting, which diminishes its external validity. However, it should be noted that this is a novel study, making it difficult to undertake a more complicated study design at this stage. One may critique that the PS always evaluated patients first and reviewed with the OSs. This pre-set order of patient evaluation was chosen to more accurately simulate the reality of what would be encountered in these types of clinics. Furthermore, the OSs’ clinical decisions were considered the gold standard and OSs in teaching hospitals are used to reviewing cases with their students, making it unlikely they would be biased negatively. The data collection method for additional management recommendations was also a limitation of the study. The investigation and treatment decisions of each clinician were simply tallied and compared between clinicians. Further investigation should be done to assess the complex clinical reasoning process underpinning the decisions.

## Conclusion

The graduating physiotherapy student and the orthopaedic surgeons made similar diagnoses and triage recommendations suggesting that a lengthy clinical experience alone is not a prerequisite for physiotherapists to help increase accessibility to orthopaedic care. Thus, a proper training in orthopaedic medicine is probably at least as or more important than clinical experience for success as an advanced practice physiotherapist.

## Data Availability

The datasets used and/or analysed during the current study are available from the corresponding author on reasonable request.

## Abbreviations

95% CI: 95% confidence interval
APP: advanced practice physiotherapist
CT-scan: computed tomography scan
MRI: magnetic resonance imaging
NSAIDs: non-steroid anti-inflammatory drugs
OS: orthopaedic surgeon
PS: physiotherapy student
VSQ-9: Visit-Specific Satisfaction Instrument
κ: Cohen’s kappa
